# Nutrikinetic study of genistein metabolites in ovariectomized mice

**DOI:** 10.1371/journal.pone.0186320

**Published:** 2017-10-23

**Authors:** Da-Hye Lee, Min Jung Kim, Eun-Ji Song, Jin Hee Kim, Jiyun Ahn, Young-Do Nam, Young-Jin Jang, Tae-Youl Ha, Chang Hwa Jung

**Affiliations:** 1 Research Group of Metabolic Mechanism, Korea Food Research Institute, Seongnam, Republic of Korea; 2 Department of Food Biotechnology, Korea University of Science and Technology, Seongnam, Republic of Korea; 3 Research Group of Gut Microbiome, Korea Food Research Institute, Seongnam, Republic of Korea; Instituto de Investigacion Sanitaria INCLIVA, SPAIN

## Abstract

This study was designed to evaluate the effect of ovariectomy on nutrikinetics of genistein metabolites. To characterize the time-dependent changes in genistein metabolite concentrations, we identified 13 genistein metabolites using ultra-performance liquid chromatography-quadrupole time-of-flight mass spectrometry. The nutrikinetics of the individual metabolites at different time points were analyzed. Nutrikinetic analysis showed that genistein, genistein 4′-glucuronide, genistein 7-glucuronide, 3-hydroxygenistein, and hippuric acid showed relatively high bioavailability in the sham group compared to that in the ovariectomy group, suggesting that ovariectomy likely results in lower genistein bioavailability. These results may be related to alteration of gut microbiota by ovariectomy. The relative abundance of species of the Parabacteroides, *Dorea*, and *Butyricimonas* genera, and Desulfovibrionaceae_unclassified, Lachnospiraceae_unclassified, and Rikenellaceae_unclassified families increased in the ovariectomy group while the relative abundance of 523_7_unclassified and Y52_unclassified_unclassified increased in the sham group. These results suggest that gut microbiota alteration by ovariectomy may affect genistein bioavailability.

## Introduction

Soybean isoflavones such as genistin and daidzin have attracted interest for a long time owing to their potential effects on bone loss [1]. Genistein is the aglycone of genistin, which is deglycosylated by intestinal glucosidases, leading to the release of the aglycone, which is absorbed and further metabolized into numerous specific metabolites including glucurono- and sulfo-conjugates [2, 3]. Currently, more than 20 genistein metabolites have been identified in animals and humans following genistein supplementation [2, 4, 5]. To improve our understanding of the bioavailability of genistein for maintaining human health, it may necessary to evaluate the nutrikinetics of individual genistein-derived metabolites, which may exert direct or indirect biological effects [4].

While previous pharmacokinetic studies of genistein have determined the phase II metabolites after enzymatic hydrolysis [6], only a few studies have analyzed genistein and its metabolites in plasma after its oral administration [2, 5]. Although genistein has low oral bioavailability [4, 7, 8], numerous studies have demonstrated a wide variety of biological activities [9]. It is possible that genistein metabolites could directly contribute to its beneficial effects. Although phase II metabolites are major genistein metabolites, genistein is metabolized by gut microbiota, leading to the release of numerous different metabolites into the systemic circulation. Dihydrogenistein and 2(4-hydroxy phenyl) propionic acid in blood have been identified as major gut-mediated metabolites, at comparably high levels to those of major phase II metabolites in blood [2].

The bioavailability of phytochemicals can be affected by many variables including age, sex, physical activity, genetic phenotype, stress, and bariatric surgery [10–12]. Estrogen deficiency is one of the most important factors contributing to osteopenia and it results in various intestinal environmental changes such as alteration of gut microbiota [13]. Although estrogenic hormonal imbalance may be affected by nutrient bioavailability [14, 15], there is a dearth of studies on the effect of physical ovariectomy (OVX) and subsequent hormonal imbalance on the bioavailability of phytochemicals such as genistein remain. One hypothesis is that OVX-induced hormonal imbalance affects gut microbiota populations, thereby affecting the bioavailability of phytochemicals such as genistein. A study has shown that the gut microbiota can affect the pharmacokinetics and bioavailability of orally administered drugs [16].

In this study, we analyzed the nutrikinetic profiles of genistein-derived metabolites in the serum of mice subjected to sham or OVX surgeries after oral administration of genistein to determine whether OVX affects genistein bioavailability. We also used 16S rRNA gene-based amplicon Iontorrent PGM sequencing of fecal matter from sham- or OVX-operated mice to determine the effects of OVX on the gut microbiota population.

## Materials and methods

### Reagents and chemicals

High-performance liquid chromatography (HPLC) grade solvents were purchased from Fisher Scientific (Pittsburgh, PA, USA). Formic acid and ammonium acetate were purchased from Merck (Darmstadt, Germany). Genistein (G6776), hippuric acid (HA, 112003), 3,4-dihydroxypenylacetic acid (DOPAC, 11569), benzoic acid (BA, 242381), and 2-(4-hydroxy phenyl)propionic acid (PA, 91378) were purchase from Sigma-Aldrich (St Louis, MO, USA). Genistein 4′-glucuronide (G-4′G, G349990), genistein 7-glucuronide (G-7G, G350015), genistein 7′-sulfate (G-4′S, G350045), and genistein 7-sulfate-4′-glucuronide (G-7S-4′G, G350050) were purchased from Toronto Research Chemicals (Toronto, Ontario, Canada). Equol 7-sulfate (E-7S, sc-219698) and dehydrogenistein (DHG, sc-498873) were purchased from Santa Cruz (Santa Cruz, CA, USA). 3-Hydroxydaidzein (3′-OHD, 1309) and 3-hydroxygenistein (3′-OHG, CFN99257) were purchased from Extrasynthese (Genay, France) and Chemface (Wuhan, China), respectively.

### Animal experiment

Female sham-operated and OVX mice (C57BL/6, 9-week old) were purchased from Central Laboratory Animal Inc., (SLC. Inc., Japan) and housed under standard laboratory conditions (temperature, 22 ± 2°C) with a 12-h light-dark cycle (7:00–19:00) with free access to food (AIN-93G diet) and water. The animal studies were approved by the Animal Care and Use Committee of the Korea Food Research Institute (KFRI-M-16014). After 8 weeks, the sham and OVX mice were administered genistein (5 mg/kg body weight in 200 μL polyethylene glycol) via gavage. After the oral administration of a single dose of genistein, blood samples were collected via cardiac puncture under anesthesia (300 mg/kg tribromoethanol intraperitoneally, IP) at different time points (0.5, 1, 2, 4, 8, and 16 h, n = 6/time point). At 0 h, the mice received only the vehicle (n = 4). Blood samples were immediately centrifuged at 2000 × *g* for 10 min, the supernatants were collected, and stored then at -80°C until the analysis. Stool samples were collected 0, 4, and 8 weeks before oral administration of genistein and stored at -80°C until used. Detailed flowcharts of the animal experiments are shown in S1 Fig.

### Sample preparation and liquid chromatography-tandem mass spectrometry (LC-MS/MS) analysis

Acetonitrile (700 μL) was added to serum samples (300 μL), the mixture was sonicated for 15 min on ice, and then centrifuged at 15,000 × *g* for 10 min. The supernatant was completely dried using a speed-vac, the residue was resuspended in methanol, filtered, and then transferred to the LC-MS/MS system. An Acquity ultra-performance-LC (UPLC) system (Waters, Milford, MA, USA) with an Acquity UPLC BEH C18 column (2.1 × 100 mm, 1.7-μm) and Waters Synapt G2-Si mass spectrometer (Waters, Manchester, UK) operating in the electrospray ionization (ESI) mode were used for the analysis. The ESI source was set in the negative ESI mode with a scan range of 50–1000 *m/z*. The mobile phases A and B, which were composed of 10 mM ammonium acetate and acetonitrile, respectively (both containing 0.1% formic acid) were run on a gradient elution program of 0–14 min, 2–95% B, with a flow rate of 0.35 mL/mL with a 5 μL injection volume. Argon and nitrogen were used as the collision and desolvation gas, respectively. The capillary voltage and cone energy voltages were set at 1.0 and 40 V, respectively. The desolvation and cone gas flow rates were 800 and 0 L/h, respectively. The source and desolvation gas temperatures were 110 and 350°C, respectively. All the spectral data were collected in continuum format using the MS^E^ acquisition mode. The mass accuracy was calibrated using sodium formate while leucine encephalin ([M-H]^-^: m/z 554.2615) was used as the lock mass. The concentration of leucine encephalin was 2 ng/mL, the flow rate was set at 5 μL/min, and the data were collected in the rage of 50–1000 *m/z*.

### Identification of genistein metabolites in serum

The genistein metabolites were identified using the UNIFI software (ver. 1.7.1, Waters) using MS-based metabolomics profiling. The peak data were collected using the mass accuracy (ppm < ± 5); fragment match tolerance, 2.0 mDa; retention time tolerance, 0.1 min; MS tolerance, 100 Da; and retention time window, 0.2 min. The analytical validation was performed by comparing the corresponding accurate masses and retention times to those of the reference compounds.

## Non-compartmental serum kinetic analysis

Non-compartmental pharmacokinetics analysis was performed for each genistein metabolite, including the evaluation of the maximum peak area (P_max_), time to reach Pmax (t_max_), terminal elimination half-time (t_1/2_), and the area under the metabolite peak versus time curve from time 0–16 h (AUC_0-16h_), using the PK solution software version 2.0 (Summit Research Services, Montrose, CO, USA).

## 16s rRNA gene amplification and DNA sequencing

Stool samples were collected at week 0, 4, and 8. The total metagenomic DNA was amplified using polymerase chain reaction (PCR) using primers targeting the V1-2 regions of the bacterial 16s rRNA gene. The primers were designed based on bacterial universal primers, and we used a modification of the 27F and 342R primers [17] fused to an eight-nucleotide barcode sequence. The PCR was performed using a C1000 Thermal Cycler (Bio-Rad, USA) and Maxime PCR PreMix (INtRON Biotechnology, Korea). The following amplification conditions were used: one cycle at 95°C for 2 min, 29 cycles at 95°C for 1 min, 58°C for 30 s, and 72°C for 30 s, followed by 72°C for 10 min. The PCR products were purified using the LaboPass PCR purification kit (Cosmo Genetech, Korea). The DNA quantity and quality were assessed using the Nanodrop ND-1000 Spectrophotometer (Thermo Scientific, Wilmington, DE, USA). Equal amounts of each sample were pooled to a total amount of 1000 ng, the DNA library was constructed using the Ion Xpress Plus Fragment Library kit (Thermo Scientific) according to the manufacturer’s instructions, and the final library was quantified using a Bioanalyzer 2100 (Agilent Technologies, Inc., Santa Clara, CA, USA) with high-sensitivity DNA chips. Emulsion PCR was performed using the Ion OneTouch 400 Template kit (Thermo Scientific) following the manufacturer’s instructions. Library sequencing was performed on a 318-chip using the Ion Sequencing 400 kit and the Ion Torrent PGM system (Thermo Scientific) according to the manufacturer’s instructions.

### Bioinformatic analysis

Raw sequences were filtered using PRINSEQ to remove low-quality or overly short sequences [18]. Sequences with corresponding barcodes and primer sequences were selected using the split_libraries.py in the quantitative insights into microbial ecology (QIIME) pipeline [19]. The errors were corrected using the Acacia tool [20]. The resulting sequences were subjected to USEARCH software analysis based on the UCHIME algorithm for the removal of chimera sequences [21, 22]. Operational Taxonomic Units (OTUs) were identified using the pick_open_reference_otus.py in the QIIME with a 97% similarity threshold and minimum cluster size of 3. All samples were subsampled to an even number of sequences for further analysis. Alpha diversity, including the observed number of OTUs and the weighted UniFrac distance between samples, was calculated using core_diversity_analysis.py in the QIIME. The PCoA plots from the weighted UniFrac metrics wer visualized using EMPeror [23]. Greengenes (http://greengenes.lbl.gov) 16S rRNA reference sequence databases was used for taxonomy assignments. The linear discriminant analysis (LDA) effect size (LEfSe) pipeline was used for biomarker discovery and to determine the taxa that best characterized each population [24]. The LDA score measured the effect size of each differentially abundant trait. Alpha values of 0.05 were used for the factorial Kruskal-Wallis test and the pairwise Wilcoxon test while a threshold of 2.0 was used for discriminative features in the logarithmic LDA score.

### Statistical analysis

Descriptive statistics were calculated for the P_max_, t_max_, t_1/2_, and AUC between the sham and OVX groups. The results were analyzed using a two-way analysis of variance (ANOVA) followed by Bonferroni post-hoc test (GraphPad Software Inc.), with a significance level of p < 0.05. Wilcoxon signed-rank test was performed using GraphPad Software to compare the nutrikinetic profiles of the sham and OVX groups.

## Results

### Identification of genistein metabolites

Isoflavones are biotransformed into various metabolites by intestinal and hepatic enzymes, thereby releasing numerous different metabolites into the systemic circulation. Genistein was administered to sham and OVX mice, and blood samples were collected at different times (0.5, 1, 2, 4, 8, and 16 h). To identify the isoflavone metabolites in the blood, the samples collected from each group over time were pooled and analyzed. Furthermore, we identified genistein metabolites by pooling multiple samples collected from the sham and OVX group using UPLC-Q-TOF-MS combined with the UNIFI software (Waters). Thirteen metabolites were detected with a mass accuracy of -0.32 mDa and, in most cases, as specific mass fragments or co-eluted with authenticated standards or both (Table 1). Two phase I and four phase II metabolites were detected in the pooled serum samples. The metabolites were in good agreement with previously published results [2, 3, 25]. Representative gut-mediated metabolites of genistein are converted to equol, which is mainly present in circulation as equol 7-sulfate. In addition, dihydrogenistein, HA, 3,4-dihydroxyphenylacetic acid, benzoic acid, and 2-(4-hydroxy phenyl)propionic acid were detected.

**Table 1:** Identified serum genistein metabolites.

No.	Metabolite group	Metabolite	RT (min)	Exact mass (m/z)	Actual mass (m/z)	Mass error (mDa)	MS Fragments
1	Intact isoflavone	Free genistein	7.12	269.0462	269.0455	-0.70	224, 206, 134
2	Phase I metabolites	3-Hydroxydaidzein	4.08	269.0450	269.0445	-0.5	241, 213, 195, 134
3		3-Hydroxygenistein	5.02	285.0399	285.0391	-0.80	255, 241, 229, 134
4	Phase II metabolites	Genistein 4′-glucuronide	4.72	445.0774	445.0765	-0.90	300, 269, 206, 134
5		Genistein 7-glucuronide	4.07	445.0774	445.0765	-0.90	300, 269, 206, 134
6		Genistein 4′-sulfate	6.19	349.0018	349.0026	0.80	269, 206
7		Genistein 7′-sulfate-4-glucuronide	3.46	525.0339	525.0337	-0.20	445, 349, 269
8	Gut-mediated metabolites	Equol 7-sulfate	2.31	321.0426	324.0438	1.21	206, 178, 162
9		Dihydrogenistein	7.34	271.0612	271.0606	-0.60	269, 165
10		Hippuric acid	1.65	178.0532	178.0513	-1.90	134, 132, 102, 77
11		3,4-Dihydroxyphenylacetic acid	0.45	167.0344	167.0363	1.90	137, 123, 108
12		Benzoic acid	1.98	121.0290	121.0277	-1.30	77
13		2-(4-Hydroxy phenyl)propionic acid	2.64	165.0564	165.0561	-0.30	136, 119

RT: retention time (min), The genistein metabolites were unambiguously identified by mass spectral and retention time matching with standard.

### Nutrikinetic analysis of genistein metabolites

**Fig 1:**
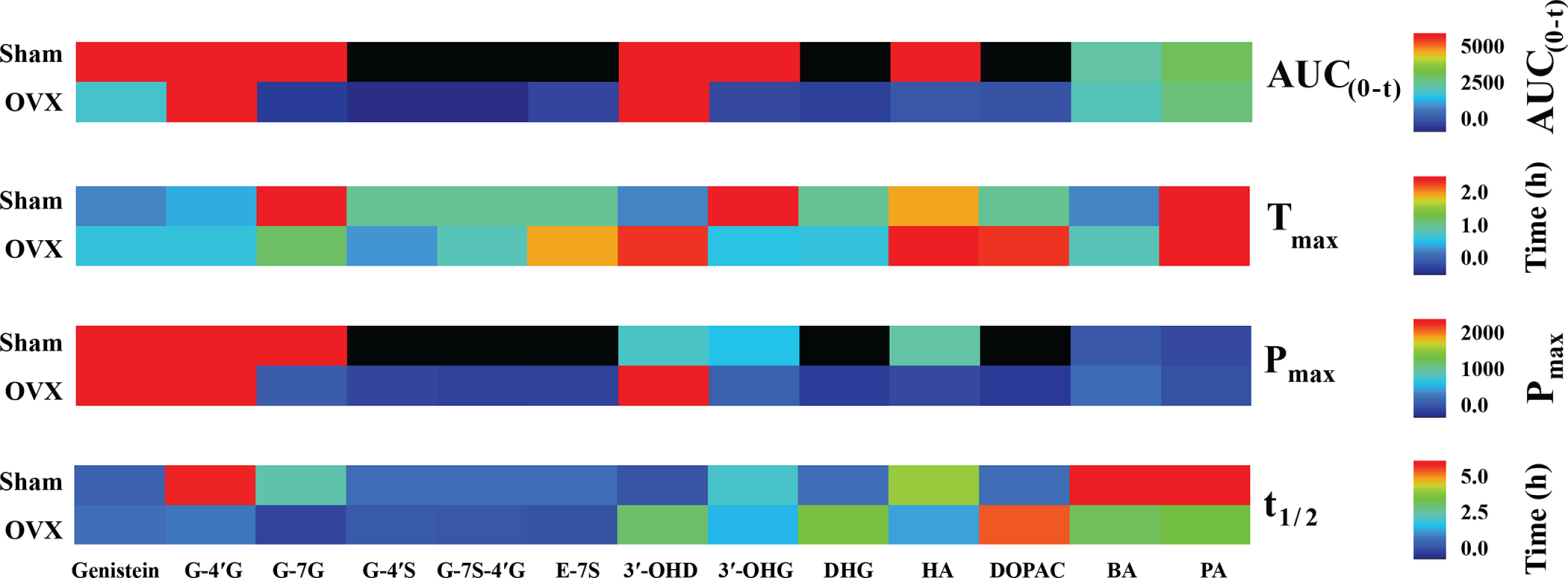
Area under the metabolite peak versus time curve (AUC), time to achieve maximum concentration (t_max_), maximum peak area (P_max_), and elimination half-life (t_1/2_) of 13 genistein metabolites in sham and OVX mice after genistein treatment. G-4′G: Genistein 4′-glucuronide; G-7G: genistein 7-glucuronide; G-7S-4′G: genistein 7-sulfate-4′-glucuronide; G-4′S: genistein 4′-sulfate; E-7S: equol 7-sulfate; 3′-OHD: 3-hydroxydaidzein; 3′-OHG: 3-hydroxygenistein; DHG: dehydrogenistein; HA: hippuric acid; DOPAC: 3,4-dihydroxypenylacetic acid; BA: benzoic acid; PA: 2-(4-hydroxy phenyl)propionic acid. Black color indicates that no linear relationship was observed between terminal semi-logarithmic intensities and values were excluded from the heatmap.

To elucidate the mechanisms of action of genistein and its role in disease prevention, it was crucial to characterize its nutrikinetic profiles as well as that of its metabolites to clarify the bioavailability. We analyzed the nutrikinetics of the identified genistein metabolites using the PK solution 2.0 software to perform a non-compartmental analysis of the P_max_, t_max_, the AUC from 0–t h (AUC_0–t_), and t_1/2_, and the values for all metabolites in both the sham and OVX groups are shown in Fig 1 and S1 Table. The pharmacokinetic profiles of genistein metabolites in the sham and OVX mice are presented in Table 2, which shows significant differences between the groups for the P_max_ of genistein, genistein-4′-glucuronide, genistein 7-glucuronide, 3-hydroxygenistein, and HA. The AUC values for genistein, genistein-4′-glucuronide, genistein 7-glucuronide, 3-hydroxygenistein, and HA were significantly higher in the sham group than they were in the OVX group. These results suggest that OVX may affect genistein bioavailability after ingestion. OVX results in various environment changes that affect absorption and metabolism. One potential mechanism for our findings may be an estrogen-mediated change in the gut microbial community.

**Table 2:** Wilcoxon signed-rank test comparison of nutrikinetic parameters of sham and ovariectomized (OVX) mice.

No.	Metabolites	P_max_	t_max_ (h)	AUC_(0–16)_	t_1/2_
1	Free genistein	P < 0.001*		P < 0.001*	
2	Genistein 4′-glucuronide	P < 0.001*		P < 0.001*	P < 0.001*
3	Genistein 7-glucuronide	P < 0.001*		P < 0.001*	P < 0.01*
4	Genistein 4′-sulfate				
5	Genistein 7-sulfate-4′-glucuronide				
6	Equol 7-sulfate				
7	3-hydroxydaidzein				P < 0.001
8	3-Hydroxygenistein	P < 0.001*	P < 0.001*	P < 0.001*	
9	Dihydrogenistein		P < 0.05*		P < 0.001
10	Hippuric acid	P < 0.05*		P < 0.001*	P < 0.01*
11	3,4-Dihydroxyphenylacetic acid	P > 0.05	P < 0.01		P < 0.001
12	Benzoic acid				P < 0.001*
13	2-(4-Hydroxy phenyl)propionic acid				P < 0.001*

* Metabolites that significantly increased in sham group compared with OVX group.

AUC, area under the metabolite peak versus time curve; T_max_, time to achieve maximum concentration; P_max_, maximum peak area; t_1/2_, elimination half-life.

### Influence of OVX on gut microbiome

**Fig 2:**
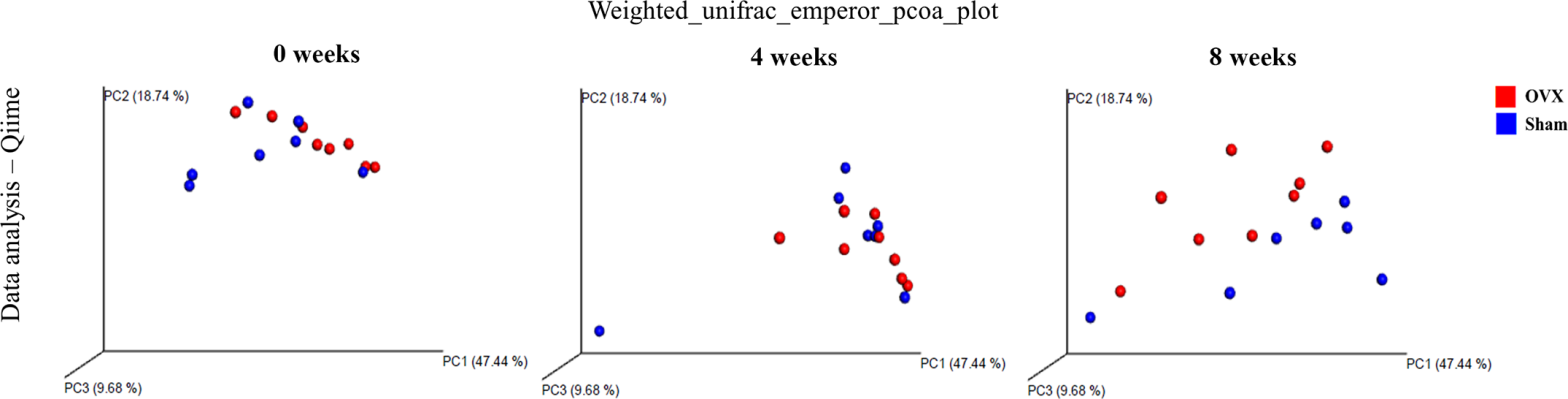
Changes in fecal microbiota over time after ovariectomy (OVX). Three-dimensional principal coordinate (PC) analysis (PCoA) based on the weighted UniFrac distance between sham and OVX groups.

Growing evidence suggests that gut microbes influence host fermentation, reduction of nitrates and sulfates, esterification, aromatic fission, hydrolysis, and deconjugation. Several host metabolic pathways are controlled by gut microbes, many of which are involved in carbohydrate or amino acid metabolism or xenobiotic biodegradation [26]. We examined the effect of OVX on gut microbiota population. The microbiota compositions of fecal samples were determined using 16S rRNA pyrosequencing. The species diversity of each group was confirmed based on the number of observed OTUs. Weighted UniFrac PCoA plotting was used to compare the microorganism types, and it showed a clear distinction between the sham and OVX group communities at week 8 (Fig 2).

**Fig 3:**
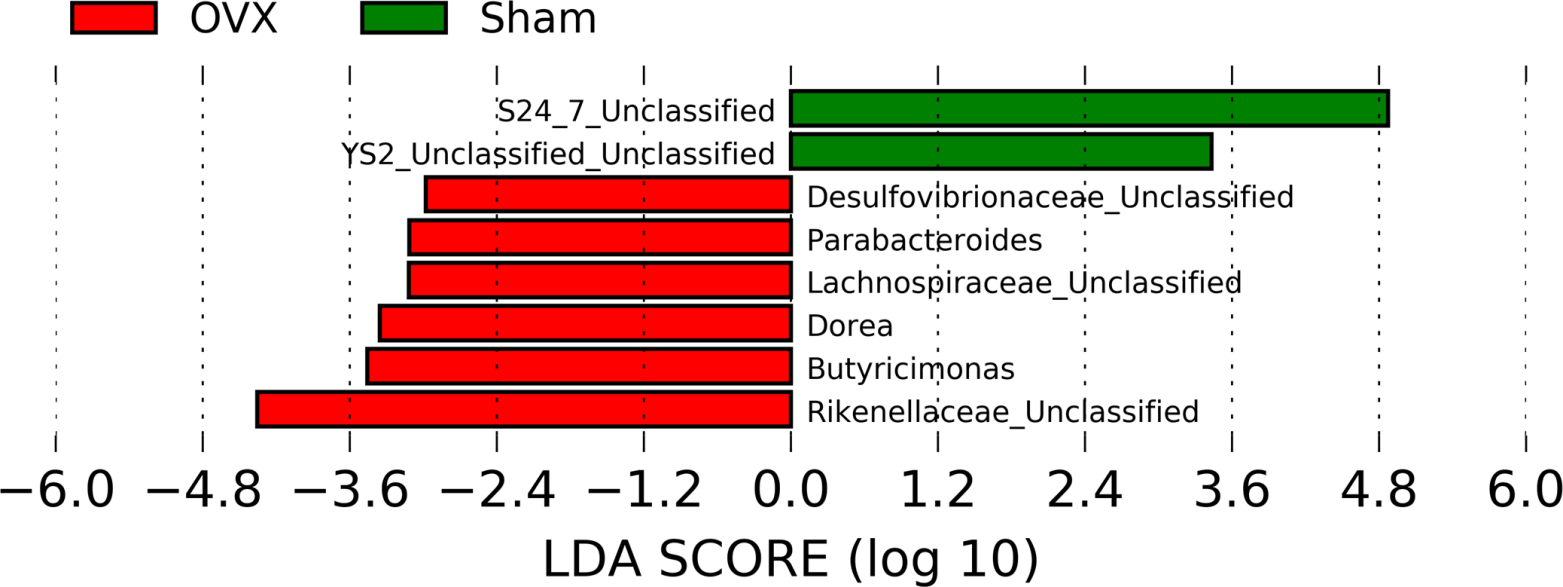
Identification of significantly different representations between samples based on the linear discriminant analysis (LDA) and effect size (LEfSe) pipeline. Genus levels with LDA scores > 2 are shown.

The LEFSE revealed the key phylotypes for the difference between the sham and OVX groups at the genus level (Fig 3). Unclassified genera of families S24_7 and orders YS2 were the most abundant in the sham group, and *Butyricimonas*, *Dorea*, *Parabacteroides*, and bacteria in the unclassified genera of the Rikenellaceae, Lachnospiraceae, and Desulfovibronaceae families were the most abundant in OVX group. This dominance was observed in all group members, and therefore, these dominant genera contributed to the differences between the sham and OVX groups and was possibly related to the progression of the effects of OVX. The data support the notion that the OVX group had a unique microbial community compared to that of the sham group. Since the gut microbiota affects the bioavailability of nutrients [27], these OVX-related differences might affect the metabolism and bioavailability of genistein.

## Discussion

In this study, we identified 13 genistein metabolites and performed nutrikinetics analysis of individual metabolites. The majority of circulating genistein is in the form of glucuronides, and a small fraction is sulfated or unconjugated [28, 29]. Although it is generally believed that aglycones are the most active compounds, conjugates have also been postulated to show biological activities [5, 30]. However, conjugates are not believed to be active species, although studies have shown the limited activity of glucuronidated genistein [31]. Conversely, several phase I genistein metabolites, which are mainly hydroxylated such as hydroxygenistein, have shown activity [32]. Phase I metabolism of genistein is an attractive target for study since the phase I biotransformation may generate metabolites that are more potent than the parent compounds [33]. Indeed, 3-hydroxydaidzein (3′-OHD) and 3- hydroxygenistein (3′-OHG) have shown excellent antioxidant and anticancer activities attributable to their structural *ortho*-dihydroxyl groups [34, 35]. Nutrikinetics analysis showed that the serum concentration of 3′-OHG was much higher in the sham mice than it was in the OVX mice. Moreover, genistein, genistein 4-glucuronide, genistein 7-glucuronide, and HA showed higher bioavailability in sham mice than in OVX mice, suggesting that OVX affected the bioavailability of genistein.

There is accumulating evidence to suggest that the gut microbiota plays a significant role in the metabolism, bioavailability, and bioactivity of dietary compounds [36, 37]. A portion of ingested genistein is not hydrolyzed or absorbed in the intestine, but rather reaches the colon where it is metabolized by gut microbiota to form colonic-derived metabolites with various reported bioactivities [38, 39]. The gut microbiota converts daidzein and genistein to equol, which has emerged as a bioactive compound with potential beneficial effects for bone health [40]. Most of the circulating equol is present as glucuronidated or sulfated conjugates, which exert estrogen agonist activity [30, 41]. Our present results indicate that equol was sulfated and not glucuronidated. Although the nutrikinetic analysis did not measure linear relationships between the terminal semi-logarithmic intensities, the equol 7-sulfate was higher in the sham group than it was in the OVX group in pooling samples (data not shown). It can be assumed that OVX changed the intestinal microbial environment.

Estrogen deficiency can cause a variety of intestinal environmental changes such as alteration of internal microbiota [42]. Our results showed that intestinal microbial changes occurred 8 weeks after the OVX. Consequently, the nutrikinetics of the genistein metabolites were analyzed in the serum after a single oral administration of genistein. The result confirmed that OVX affected the bioavailability of genistein. OVX can affect the bioavailability of phytochemicals by inducing gut microbial and environmental changes [13], which subsequently affect the metabolism and absorption of phytochemicals. After 8 weeks, *Parabacteroides*, *Dorea*, and *Butyricimonas* were the most abundant in the OVX group. *Parabacteroides* was found at higher levels in patients with nonalcoholic steatohepatitis [43], and *Dorea* showed a higher abundance in irritable bowel syndrome [44], which indicated an association between gut microbiota and disease. OVX appear to have deleterious effects on the intestinal environment. However, *Parabacteriodes* and *Butyricimonas* are also known to enhance the secretion of anti-inflammatory cytokines that inhibit inflammation, and therefore, the gut microbiota is shifted toward an increased abundance of beneficial anti-inflammatory bacteria [45]. Therefore, the correlation between gut microbiota and bioavailability of this study is limited. However, the loss of community diversity can be explained by the OVX-induced dysbiosis in the gut microbiota community. In future, the relationship between intestinal microorganisms and genistein metabolism and absorption needs to be studied more precisely.

Our results demonstrated the significant differences in the nutrikinetic characteristics of genistein between the sham and OVX groups. These differences may depend on variations in the gut microbiota. OVX alters the gut environment and, thus, may affect genistein bioavailability. Further investigation would be needed to determine whether these microbial environmental changes are regulated by food consumption.

## Supporting information

S1 Fig Study design. Detailed flow chart of animal experiments. After 2 weeks of recovery from ovariectomy, the experiment was started and the time was 0 week. Stool was received at 4 weeks and 8weeks that was before genistein administration, respectively. Blood samples were collected at different time points (0.5, 1, 2, 4, 8, and 16 h) after genistein ingestion. Blood samples were analyzed using UPLC/2-TOF-MS and Non-compartmental pharmacokinetics analysis was performed for each identified genestein metabolites.
(TIF)

S1 Table Nutrikinetic parameters of genistein-derived metabolites. AUC, area under the metabolite peak versus time curve; T_max_, time to achieve maximum concentration; P_max_, maximum peak area; t_1/2_, elimination half-life.
(DOCX)
