# Supplementary figures and images for: Nutrikinetic study of genistein metabolites in ovariectomized mice
Source: PLoS One. 2017 Oct 23;12(10):e0186320. doi: 10.1371/journal.pone.0186320 (PMC5653299; doi:10.1371/journal.pone.0186320)

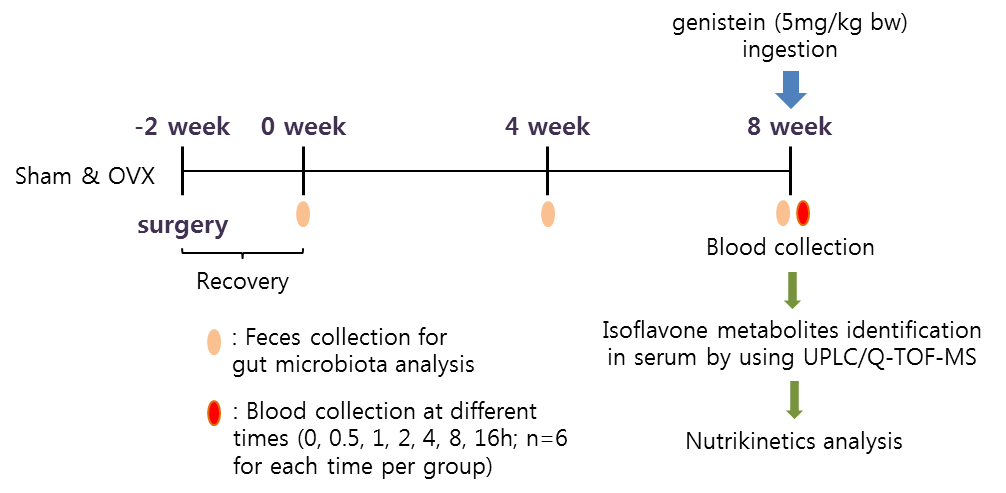

Supplement: S1 Fig — Detailed flow chart of animal experiments. After 2 weeks of recovery from ovariectomy, the experiment was started and the time was 0 week. Stool was received at 4 weeks and 8weeks that was before genistein administration, respectively. Blood samples were collected at different time points (0.5, 1, 2, 4, 8, and 16 h) after genistein ingestion. Blood samples were analyzed using UPLC/2-TOF-MS and Non-compartmental pharmacokinetics analysis was performed for each identified genestein metabolites. (TIF) [file pone.0186320.s001.tif]
