# Supplementary material for: Nutrikinetic study of genistein metabolites in ovariectomized mice
Source: PLoS One. 2017 Oct 23;12(10):e0186320. doi: 10.1371/journal.pone.0186320 (PMC5653299; doi:10.1371/journal.pone.0186320)
Supplement: S1 Table — AUC, area under the metabolite peak versus time curve; Tmax, time to achieve maximum concentration; Pmax, maximum peak area; t1/2, elimination half-life. (DOCX) [file pone.0186320.s002.docx]

| **S1 Table. Nutrikinetic parameters of genistein-derived metabolites.** | | | | | | | | | |
| --- | --- | --- | --- | --- | --- | --- | --- | --- | --- |
| No. | Metabolites | P_max_ | | t_max_ (h) | | AUC (0-t) | | t_1/2_ | |
|  |  | Sham | OVX | Sham | OVX | Sham | OVX | Sham | OVX |
| 1 | Free genistein | 29757 ± 1454 | 5270 ± 607 | 0.5 ± 0 | 0.75 ± 0.25 | 124968 ±20537 | 1986 ± 25 | 0.77 ± 1.02 | 1.00 ± 0.02 |
| 2 | Genistein 4′-glucuronide | 59431 ± 1680 | 2615 ± 586 | 0.63 ± 0.22 | 0.75 ± 0.25 | 189737 ± 10063 | 6469 ± 383 | 4.81 ± 0.15 | 1.13 ± 0.08 |
| 3 | Genistein 7-glucuronide | 4566 ± 444 | 285 ± 20 | 2.05 ± 0.09 | 1.13 ± 0.22 | 35777 ± 3935 | 274 ± 37 | 2.30 ± 0.22 | 0.41± 0.20 |
| 4 | Genistein 4′-sulfate | - | 168 ± 67 | - | 0.55 ± 0.05 | - | 63 ± 17 | - | 0.67 ± 0.29 |
| 5 | Genistein 7-sulfate-4′-glucuronide | - | 135 ± 62 | - | 0.88 ± 0.22 | - | 70 ± 10 | - | 0.62 ± 0.28 |
| 6 | Equol 7-sulfate | - | 145 ± 25 | - | 1.63 ± 0.65 | - | 413 ±42 | - | 0.56 ± 0.10 |
| 7 | 3-hydroxydaidzein | 810 ± 100 | 1999 ± 36 | 0.5 ± 0 | 1.88 ± 0.22 | 15809 ± 13300 | 7081 ± 96 | 0.58 ± 0.07 | 2.77 ± 0.56 |
| 8 | 3-Hydroxygenistein | 708 ± 10 | 323 ± 42 | 4.39 ± 2.71 | 0.73 ± 0.18 | 5699 ± 409 | 485 ± 59 | 2.00 ± 0.18 | 1.63 ± 0.11 |
| 9 | Dihydrogenistein | - | 115 ± 37 | - | 0.75 ± 0.25 | - | 315 ± 17 | - | 3.17 ± 0.62 |
| 10 | Hippuric acid | 945 ± 66 | 199 ± 10 | 1.63 ± 0.22 | 2.5 ± 0.35 | 4891 ± 107 | 644 ± 160 | 3.47 ± 0.48 | 1.49 ± 1.09 |
| 11 | 3,4-Dihydroxyphenylacetic acid | - | 87 ± 9 | - | 1.88 ± 0.22 | - | 567 ± 58 | - | 4.50 ± 0.41 |
| 12 | Benzoic acid | 26 2 ± 38 | 397 ± 39 | 0.5 ± 0 | 0.88 ± 0.41 | 2358 ± 217 | 2163 ± 178 | 5.23 ± 0.23 | 2.89 ± 0.11 |
| 13 | 2-(4-Hydroxy phenyl)propionic acid | 19 8± 2 | 218 ± 20 | 8.0 ± 0.61 | 7.13 ± 0.54 | 2863 ± 158 | 2680 ± 520 | 5.15 ± 0.84 | 3.03 ± 0.56 |
